# Exposure to the Insecticide Sulfoxaflor Affects Behaviour and Biomarkers Responses of *Carcinus maenas* (Crustacea: Decapoda)

**DOI:** 10.3390/biology10121234

**Published:** 2021-11-26

**Authors:** Jadilson M. Damasceno, Lénia D. Rato, Tiago Simões, Inês F. C. Morão, Gabriela Meireles, Sara C. Novais, Marco F. L. Lemos

**Affiliations:** 1 MARE-Marine and Environmental Sciences Centre, ESTM, Polytechnic of Leiria, 2520-630 Peniche, Portugal; jadilson.damasceno@ipleiria.pt (J.M.D.); lenia.rato@ipleiria.pt (L.D.R.); tiago.simoes@ipleiria.pt (T.S.); ines.morao@ipleiria.pt (I.F.C.M.); gabriela.meireles@ipleiria.pt (G.M.); 2 Department of Coastal Systems, NIOZ-Royal Netherlands Institute for Sea Research, ’t Horntje, 1797 SZ Texel, The Netherlands

**Keywords:** coastal invertebrates, crab, ecotoxicology, energy metabolism, neurotoxicity, oxidative stress, pesticide

## Abstract

**Simple Summary:**

Sulfoxaflor is an insecticide for which there are few studies regarding its toxicity to non-target organisms. The present study aimed to investigate the acute and sub-lethal effects of sulfoxaflor on *Carcinus maenas* by addressing survival, behaviour, and biomarkers. Sulfoxaflor affected feed intake and motricity of *C. maenas*. From the integrated analysis of endpoints, with the increase in concentrations of sulfoxaflor, after seven days, one can notice a lower detoxification capacity, higher lipid peroxidation, and higher motricity effects and lower feed intake. This study aims to contribute to the understanding of the negative impacts of sulfoxaflor on green crabs and increase knowledge of this pesticide toxicity to non-target coastal invertebrates.

**Abstract:**

Sulfoxaflor is an insecticide belonging to the recent sulfoximine class, acting as a nicotinic acetylcholine receptor (nAChRs) agonist. There are few studies regarding sulfoxaflor’s toxicity to non-target organisms. The present study aimed to investigate the acute and sub-lethal effects of sulfoxaflor on *Carcinus maenas* by addressing survival, behaviour (feed intake and motricity), and neuromuscular, detoxification and oxidative stress, and energy metabolism biomarkers. Adult male green crabs were exposed to sulfoxaflor for 96 h and an LC_50_ of 2.88 mg L^−1^ was estimated. All biomarker endpoints were sampled after three (T3) and seven (T7) days of exposure and behavioural endpoints were addressed at T3 and day six (T6). Sulfoxaflor affected the feed intake and motricity of *C. maenas* at T6. From the integrated analysis of endpoints, with the increase in concentrations of sulfoxaflor, after seven days, one can notice a lower detoxification capacity (lower GST), higher LPO levels and effects on behaviour (higher motricity effects and lower feed intake). This integrated approach proved to be valuable in understanding the negative impacts of sulfoxaflor on green crabs, while contributing to the knowledge of this pesticide toxicity to non-target coastal invertebrates.

## 1. Introduction

Aquatic pollution is a major concern due to its detrimental effects in biota. Agriculture is one of the main sources of water pollution through the run-off of metals, pathogens, nutrients, and pesticides [1].

Economic loss in agriculture, namely caused by insect pests, requires the application of pesticides [2]. Different classes of pesticides, each with a particular mode of action (MoA), have proven to be useful in the control of various sap-feeding insects [3,4]. However, the high production rate and indiscriminate use of these chemicals has raised concerns about the contamination of ecosystems, due to their toxicity to organisms and persistence in the environment [5,6,7].

Sulfoxaflor, methyl(oxo){1-[6-(trifluoromethyl)-3-pyridyl]ethyl}-ʎ^6^-sulfanylidene) cyanamide, is an insecticide developed by Dow AgroSciences [8], classified in the sulfoximines class, used in the control of a wide range of insect strains that have demonstrated resistance to other pesticides, including neonicotinoids [4,9,10]. Sulfoxaflor’s (Isoclast™ active) mode of action consists of an agonist interaction between pesticides with nicotinic acetylcholine receptors (nAChRs), acting as an activator [4,8] through contact or ingestion. Thus, sulfoxaflor targets the nervous system, causing excitatory symptoms, namely tremors, leg extension or curling, partial or complete paralysis and mortality, as observed in studies carried out with insects [4,11]. Due to some similarities in the mode of action between other neonicotinoids and sulfoxaflor, there are concerns about sulfoxaflor’s possible effects on non-target organisms [4,12]. Indeed, a very limited number of studies are available about sulfoxaflor non-targeted toxicity [13]. Sulfoxaflor, under field conditions in soil, has a half-life (DT_50_) average of four days. However, the DT_50_ in the sediment/aquatic systems can range between 37 and 88 days [8].

To understand how the new generation pesticide sulfoxaflor affects non-target organisms in coastal/marine environments, an ecotoxicological approach using the European green crab *Carcinus maenas* as a non-target model species was performed, recurring to biomarker responses [14]. Crustaceans, due to their wide distributions, sedentary life-style, and relative tolerance to contaminants, are model organisms commonly used to assess the quality of aquatic ecosystems, as reviewed in Lionetto et al. [14]. *Carcinus maenas* has been extensively studied for more than 45 years, being proposed as a suitable model species in ecotoxicology, while also having a significant ecological relevance [15] and being economically important in some regions, as reviewed in Klassen and Locke [16] and Young and Elliott [17].

In this context, this study investigated acute and sub-lethal exposure to sulfoximine-based insecticide sulfoxaflor on the non-target coastal *C. maenas*, interconnecting different levels of biological organisation, namely the survival, behaviour (feed intake and motricity), and biomarker responses. When a xenobiotic enters the organism’s body, detoxification mechanisms are activated. This involves two phases of biotransformation [18,19]. In Phase I, a functional group is introduced into the lipophilic contaminant chemical structure, increasing their polarity, and in Phase II small molecules, such as glutathione (GSH), are conjugated with the xenobiotics by enzymes (e.g., glutathione-S-transferase—GST), increasing their solubility for elimination [18,19,20]. By interfering with the redox cycling, this detoxification process can also potentiate the production of reactive oxygen species (ROS) [21,22,23], which is already naturally occurring due to the metabolic activity and energy production involving molecular oxygen (O_2_) [24]. The most commonly evaluated ROS are the superoxide anion (O_2−_), hydroxyl radical (HO•), hydrogen peroxide (H_2_O_2_), and singlet oxygen (^1^O_2_) [24,25]. To prevent adverse effects, there are antioxidant enzymes and low molecular weight scavengers responsible for removing these oxyradicals [22,25], such as the superoxide dismutase (SOD) enzyme, which produces H_2_O_2_ during dismutation of O_2_^−^, followed by catalase (CAT) and glutathione peroxidase (GPx) enzymes, that convert H_2_O_2_ into H_2_O [24,26,27]. Nonetheless, high quantities of ROS can overcome the defensive mechanisms and cause oxidative damage in macromolecules such as lipids, proteins, and DNA [21]. To cope with this stress, the organisms may undergo metabolic alterations and energetic trade-offs [28]. Isocitrate dehydrogenase—IDH (aerobic pathways) and lactate dehydrogenase—LDH (anaerobic pathways), are useful biomarkers for addressing alterations in the energy metabolism [29,30,31]. Cellular oxygen consumption rate can be measured through the electron transport system (ETS) activity, as energetic status information [32]. Furthermore, acetylcholinesterase (AChE), an enzyme that hydrolyses the neurotransmitter acetylcholine, is a relevant biomarker in the identification of neuromuscular toxic affects of a xenobiotic [14,33].

By addressing these multi-biological levels of biological organisation—ranging from survival to behavioural changes such as feed intake and motricity, and biochemical endpoints—the present study contribute to the knowledge on the sulfoxaflor mode of toxic action and impact on this non-targeted aquatic crustacean and provide insight into potential impacts and higher levels of biological organisation.

## 2. Materials and Methods

### 2.1. Ethical Experiments

Although not mandatory for invertebrates and considering the welfare of the animals used for experimental and scientific purposes, all experiments were conducted according to the European Union Council’s (Directive 2010/63/EU) [34] ethical guidelines and the Portuguese Ministry of agriculture, sea, environment and territory planning (Decreto-Lei 113/2013) [35].

### 2.2. Crab Sampling and Acclimation

*Carcinus maenas* adult males were collected in Óbidos lagoon, Portugal, in fishermen traps (net mesh of 20 mm). The organisms were transported in dry containers to aquaculture facilities in CETEMARES building, Peniche, for further acclimation and experimental procedures.

Size-selected green crabs, for acute and sub-lethal tests, had an average carapace length of 3.44 ± 0.18/3.63 ± 0.27 cm (mean ± SD), and wet weight of 9.29 ± 1.45/10.75 ± 2.34 g (mean ± SD), respectively. These were re-immersed in containers filled with natural filtered seawater to release ammonia [36], and to remove sediment, algae, or other detritus attached to their body, and then transferred into a recirculating aquaculture system (RAS). This RAS contained mechanical (50 µm glass fibre bags, TMC) and biological filtration (bioballs with nitrifying bacteria), along with chemical UV disinfection (V^2^ Vecton 300, TMC). Acclimation took place for a period of at least 14 days with a photoperiod set to 16 h light and 8 h dark [37], and water parameters were set as found in the Óbidos lagoon during spring: the temperature was set to 19 °C, seawater salinity between 32 and 34 ppt, pH maintained at 7.9 to 8.2, and dissolved oxygen (DO) at 6 to 8 mg L^−1^ [38]. These parameters were evaluated daily with a multiparameter probe (YSI, Professional Plus, Yellow Springs, OH, USA). Crabs were fed every other day with mussel kernels (*Mytilus edulis*). To maintain quality, water was syphoned the day after feeding, and partial water changes (30% or 60%) were performed. Organisms were not fed 24 h before the experiments, as advised in Rodrigues et al. [39].

### 2.3. Experimental Design

#### 2.3.1. Material and Pesticide Stock Solution

Before experimental bioassays, glass materials followed a washing scheme of nitric acid bath (HNO_3_ 10%) for 24 h, Extran base bath (10%) another 24 h, and then machine wash (acid, basic, and distilled water finish), to ensure clean material.

Closer^®^, Isoclast™ active (Dow AgroSciences, Sevilla, Spain), suspension concentrate containing 120 g L^−1^ or 11.3% (*w*/*w*) of sulfoxaflor (CAS number 946578-00-3, Dow AgroSciences, 2014 [8]), was stored at room temperature protected from light. The sulfoxaflor stock solution (0.6 mg mL^−1^ nominal concentration) was prepared with ultrapure water (MilliQ^®^, Merck, Darmstadt, Germany, HE, DE).

#### 2.3.2. Acute Toxicity Test

To estimate lethal concentrations, six *C. maenas*, per treatment, were exposed for 96 h to the pesticide sulfoxaflor to one of the six nominal concentrations determined through logarithmic calculations (0, 1.0, 1.6, 2.5, 4.0, 6.3, or 10 mg L^−1^ in seawater).

Green crabs were randomly exposed in individual glass flasks containing 700 mL of the exposure solution at the desired final concentration and constant aeration. Exposure solution was renewed daily. Seawater parameters were set as described above for acclimation (see Section 2.2) and verified and adjusted daily after using a multiparameter probe (YSI, Professional Plus, Yellow Springs, OH, USA) (Table S1).

#### 2.3.3. Sub-Lethal Toxicity Tests

Fifteen *C. maenas*, per treatment, were exposed to sulfoxaflor for 168 h (7 days) to one of the seven treatments determined by logarithmic calculations (0, 0.05, 0.08, 0.15, 0.27, 0.5, or 0.9 mg L^−1^). The nominal concentration range for these tests was defined according to the estimated LC_10_ from the acute exposure [1.74 mg L^−1^ (±95% CI: 0.82–2.30)], encompassing half the LC_10_ (approx.) as the higher concentration in order to minimize lethal effects.

The organisms were exposed in individual glass flasks filled with saltwater plus the necessary volume of the pesticide stock solution to meet target concentrations to 700 mL. Replicates were randomly distributed and provided with constant aeration. Exposure solution was renewed daily. Saltwater parameters were set as described above for acclimation (see Section 2.2), and verified daily using a multiparameter probe (YSI, Professional Plus, Yellow Springs, OH, USA) (Table S1). Organisms were fed *ad libitum* on days 3 and 6 with mussel kernels (*Mytilus edulis*).

On day 3 of exposure, seven replicates of each treatment were ice anaesthetised (−20 °C for 15 min), then euthanized by using a needle to perforate both hind nerve centre and front nerve centre [40] and dissected. Hepatopancreas (H), gills (G), and muscle (M) from the abdomen were collected for biochemical analysis. Tissues were stored at −80 °C prior to homogenization and further analysis.

Behavioural tests, namely feed intake and motricity, were carried out in the remaining replicates on day 3 and on day 6 of exposure, with a minimum of seven replicates per treatment (maximum *N* = 8). The experiment was performed at day 6 in order for the minor manipulation not to interfere with the endpoints addressed at day 7. Prior to testing, crabs’ wet weight and carapace length were assessed to normalize for food intake values. A motricity evaluation method was created and optimized, based on the forage activity [41] and the competition between *Carcinus maenas* and *Hemigrapsus takanoi* for food [42]. Briefly, the motricity test was performed by placing mussel kernels in the same point of the exposure containers, and then, the time taken by the crab to react and reach the food was counted, establishing 5 min as the time limit. For the feed intake, initial mussel kernels wet weight was measured, prior to the motricity test, and the organisms were fed for a 4 h period, after which the food final wet weight was measured for feed consumption quantification, adapted from Abreu at al. [43].

On day 7 of exposure, the remaining organisms (minimum of seven) were ice anaesthetised, euthanized and the tissues dissected for biochemical analysis.

### 2.4. Tissue Preparation and Biomarkers Analysis

*Carcinus maenas* tissues were homogenized in 0.1 M potassium-phosphate (K_2_HPO_4_/KH_2_PO_4_) buffer (pH 7.4) in a proportion (m:v) of 1:5 (muscle and gills) and 1:15 (hepatopancreas), using a mechanical homogenizer (Ystral, X10/25, Ballrechten-dottingen, BW, DE).

Biomarker responses after 3 and 7 days of exposure were addressed. Muscle homogenates were centrifuged at 3000× *g* during 5 min at 4 °C and the supernatant separated into different microtubes for protein quantification and the activity determination of isocitrate dehydrogenase (IDH), lactate dehydrogenase (LDH) and acetylcholinesterase (AChE). Additionally, muscle homogenates plus ETS buffer [0.1 M Tris-HCl, 15% (*w*/*v*) Poly Vinyl Pyrrolidone, 153 µM MgSO_4_, 0.2% (*w*/*v*) Triton X-100, and pH 8.5] were centrifuged at 1000× *g* for 10 min at 4 °C and the supernatant separated for electron transport system (ETS) analysis. Two extra aliquots of muscle homogenate were separated for lipid peroxidation [LPO; in 4% BHT (2,6-Di-tert-butyl-4-methylphenol) in methanol] and DNA damage (DNAd) measurements. Hepatopancreas homogenates were centrifuged at 10,000× *g* for 20 min at 4 °C and the post-mitochondrial supernatant (PMS) distributed into microtubes to measure protein content and the activity of superoxide dismutase (SOD) and glutathione S-transferase (GST). Hepatopancreas homogenates were also separated for LPO and DNAd quantification. Gill homogenates were used for quantification of reactive oxygen species (ROS), LPO, and DNAd. All homogenates and separated aliquots were stored at −80 °C until further analysis. Spectrophotometric measurements were performed in triplicates using a Synergy H1 Hybrid Multi-Mode microplate reader (Biotek^®^ Instruments, Winooski, VT, USA) and using a homogenization buffer as reaction blanks.

#### 2.4.1. Protein Quantification

Protein quantification was performed for normalization purposes following the Bradford method [44] and adapted to a 96-well microplate, using bovine ɣ-globulin (Sigma-Aldrich, Darmstadt, Germany, HE, DE) as protein standard solution. Absorbance was read at 600 nm. The results were represented in mg of protein mL^−1^.

#### 2.4.2. Energy Metabolism Related Biomarkers

Isocitrate dehydrogenase (IDH) activity was quantified following the Ellis and Goldberg [45] method and adapted to a microplate [30]. IDH, an enzyme involved with aerobic metabolism, plays a role in the decarboxylation of isocitrate (DL-isocitric acid). Consequently, the production of NADPH during this process was followed at 330 nm for 3 min (min). Results were expressed as nmol min^−1^ mg^−1^ of protein (ε = 6.22 × 10^3^ M^−1^cm^−1^). Lactate dehydrogenase (LDH) activity was assessed following the methods of Vassault [46] and Diamantino et al. [29]. LDH, an enzyme involved with anaerobic metabolism, converts pyruvate to lactate through oxidation of NADH into NAD^+^. NADH decrease is followed at 330 nm for 3 min. Results were expressed in nmol min^−1^ mg^−1^ of protein (ε = 6.22 × 10^3^ M^−1^cm^−1^). The electron transport system (ETS) activity biomarker consists of the determination of the oxygen consumption rate, through the method described by De Coen and Janssen [32], and following the built-up of the complex INT (p iodo-nitro-tetrazolium)-formazan for 3 min at 490 nm. Then, a stoichiometrical relationship (2 µmol of INT-formazan formed is equivalent to 1 µmol of oxygen consumed) was applied to determine the oxygen consumption rate. Results were presented in nmol O_2_ h^−1^g^−1^ of wet weight.

#### 2.4.3. Detoxification and Oxidative Stress Related Biomarkers

Glutathione S-transferase (GST) activity was quantified based in Habig et al. [47] method. The compound thioether is produced by the conjugation between the substrate 1-chloro-2,4-dinitrobenzene (CDNB) and reduced glutathione (GSH) in the presence of GST. The thioether formation is followed by reading the absorbance at 330 nm for 3 min. Results were expressed as nmol min^−1^ mg^−1^ protein (ε = 9.6 × 10^3^ M^−1^cm^−1^). Reactive oxygen species (ROS) levels were quantified according to Socci et al. [48] method by measuring the conversion of non-fluorescent 2′,7′-dichlorofluorescein diacetate (DCFDA) into 2′,7′-dichlorofluorescein (DCF) in the presence of ROS. DCF is a highly fluorescent compound read at 485:525 nm excitation: emission. Results were expressed in FU mg^−1^ of wet weight. Superoxide dismutase (SOD) activity quantification followed the McCord and Fridovich [49] method, adapted to microplate [30]. The protocol consists of measuring the inhibition of the reduction of cytochrome C, resulting from the competition of SOD for the superoxide radicals (O_2_^−^) produced by the xanthine/xanthine oxidase complex. The reduction of cytochrome C was observed at 550 nm for 10min. Results were expressed as U mg^−1^ of protein. Lipid peroxidation (LPO) was measured following the methods described by Bird and Draper [50] and Ohkawa et al. [51], adjusted from Torres et al. [52]. The samples were submitted to a sequence of treatments with trichloroacetic acid (TCA), Tris-HCl with DTPA, and 2-thiobarbituric acid (TBA). Following heat and centrifugation, the supernatant was used to quantify the content of thiobarbituric acid reactive substances (TBARS) at 535 nm. Results were expressed as nmol TBARS g^−1^ of wet weight (ε = 1.56 × 10^5^ M^−1^cm^−1^). DNA damage (DNAd) was determined following the DNA alkaline precipitation assay [53], adapted from De Lafontaine et al. [54]. The protocol consists of centrifuging samples to obtain the precipitation of intact DNA associated with nucleoproteins. The supernatant, containing damaged DNA (strand breaks), was mixed with Hoechst dye (1 µg mL^−1^ bis-benzimide, Sigma-Aldrich) and the fluorescence read at 360:460 nm of excitation:emission. Results were expressed as µg g^−1^ of wet weight.

#### 2.4.4. Neuromuscular Toxicity

Acetylcholinesterase (AChE) activity was measured following the Ellman et al. [55] method, adapted to a microplate [56]. AChE plays a role in catalysing the hydrolysis of acetylthiocholine, producing acetate and thiocholine. DTNB [5,5′-dithiobis-(2-nitrobenzoic acid] reacts with thiocholine, generating the yellow 2-nitro-5-thiobenzoate anion (TNB^2−^). This reaction was read at 414 nm for 5 min. Results were expressed in nmol min^−1^ mg^−1^ of protein (ε = 13.6 × 10^3^ M^−1^cm^−1^).

### 2.5. Statistical Analysis

For acute toxicity, the estimation of lethal concentrations (LCx) was performed through probit analysis. All data were checked for normality using the Shapiro–Wilk normality test (*p* > 0.05) and for homoscedasticity using Levene’s test (*p* > 0.05). The data was analysed through generalized linear modelling (GzLM, *p* < 0.05), where biomarker and behavioural responses were used as response variables, while concentration and exposure times were used as predictable factors. After the GzLM, pairwise comparisons were performed by Least Significant Difference analysis (LSD, *p* < 0.05), and model validation was carried out as in [57,58,59]. Significant positive or negative correlations between biomarkers and behavioural responses were assessed through Spearman correlations (* *p*-value < 0.05, ** *p*-value < 0.01). For the mentioned data analysis, IBM SPSS Statistic 26 software was used. Furthermore, the principal component analysis (PCA) was employed to investigate associations between biomarkers/behavioural endpoints and pesticide concentrations. Detrended correspondence analysis (DCA) was applied to determine if the lengths of gradient were <3 on the first axis, referring to linear model. PCAs were then performed on averaged biomarkers and behavioural data. DCA and PCA analyses were performed with the software CANOCO version 4.5 for windows (Biometris, Wageningen, GE, NL) [60].

## 3. Results

The estimated 96 h LC_50_ (±95% CI) for sulfoxaflor was 2.88 mg L^−1^ (2.14–4.00) and the 96 h LC_10_ was 1.74 mg L^−1^ (0.82–2.30). Other LCx values are presented in Table S2.

### 3.1. Behavioural Effects

**Figure 1:**
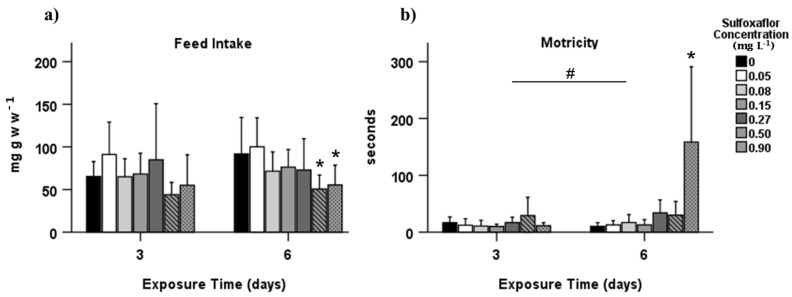
Behavioural responses of *Carcinus maenas* after 3 and 6 days of exposure to sulfoxaflor (mean ± SD): (**a**) feed intake; (**b**) motricity. * Indicates statistically significant differences in relation to control, and # denotes statistically significant differences between exposure time 3 and 6 (*p* < 0.05, GzLM, followed by the pairwise comparison method with adjustment to LSD).

Behavioural changes were not found to be significant after 3 days of exposure (*p* > 0.05). However, sulfoxaflor significantly affected the behavioural responses after 6 days of exposure, decreasing the feed intake 39.5 and 44.9%, at the two highest concentrations tested, 0.5 (*p* = 0.008) and 0.9 mg L^−1^ (*p* = 0.020), respectively (Figure 1a), and increasing the time taken to reach the food (motricity) at the highest concentration (*p* < 0.001; Figure 1b).

### 3.2. Biochemical Biomarkers

**Figure 2:**
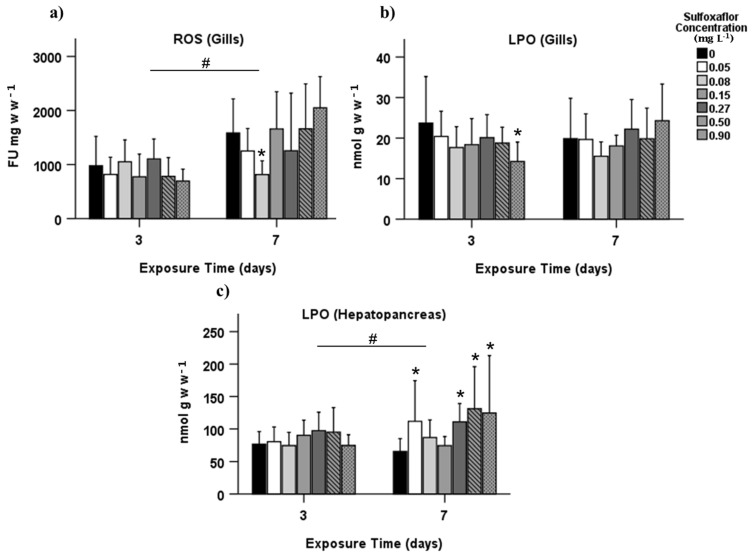
Biochemical biomarkers responses measured in gills and hepatopancreas of *Carcinus maenas* after 3 and 7 days of exposure to sulfoxaflor (mean ± SD), related to oxidative stress: (**a**) reactive oxygen species (ROS); (**b**) lipid peroxidation (LPO) measured in gills; and (**c**) LPO measured in hepatopancreas. * Indicates statistically significant differences in relation to control, and # denotes statistically significant differences between exposure time 3 and 7 (*p* < 0.05, GzLM, followed by pairwise comparison method with adjustment to LSD). Only the biomarkers with significant differences are shown.

**Figure 3:**
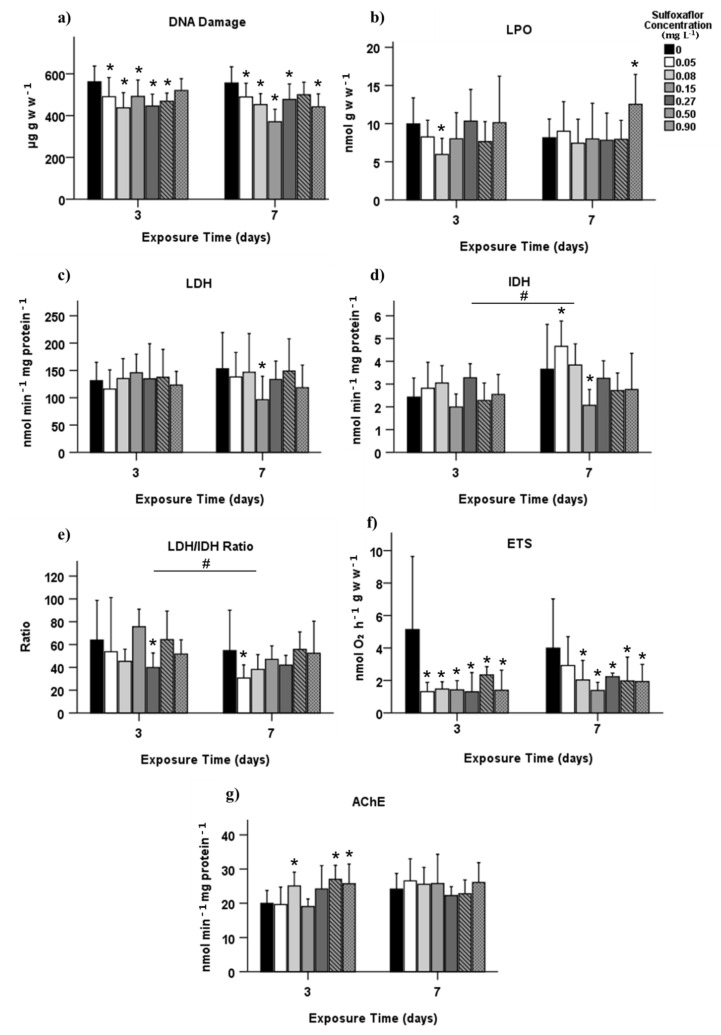
Biochemical biomarkers responses measured in muscle of *Carcinus maenas* after 3 and 7 days of exposure to sulfoxaflor (mean ± SD), related to oxidative stress—(**a**) DNA damage; (**b**) lipid peroxidation (LPO), energy metabolism—(**c**) lactate dehydrogenase (LDH); (**d**) isocitrate dehydrogenase (IDH); (**e**) LDH/IDH ratio; (**f**) electron transport system (ETS), and neuromuscular toxicity—(**g**) acetylcholinesterase (AChE). * Indicates statistically significant differences in relation to control, and # denotes statistically significant differences between exposure time 3 and 7 (*p* < 0.05, GzLM, followed by pairwise comparison method with adjustment to LSD). Only the biomarkers with significant differences are shown.

The results are here divided into each analysed tissue: gills and hepatopancreas (detoxification—GST, and oxidative stress—SOD, ROS, LPO, DNAd; Figure 2 and Figure S1), and muscle (oxidative stress—LPO, DNAd, energy metabolism—LDH, IDH, LDH/IDH ratio, ETS, and neuromuscular toxicity related—AChE; Figure 3). In the gills, no significant effects were found in reactive oxygen species (ROS) levels after three days of exposure (T3) (*p* > 0.05). However, there was a significant decrease in ROS at a concentration of 0.08 mg L^−1^ after 7 days of exposure (T7; *p* = 0.004, Figure 2a). Lipid peroxidation (LPO) from gills presented a significant reduction at the highest concentration in T3 (*p* = 0.006, Figure 2b). LPO quantified in hepatopancreas has increased at 0.05 mg L^−1^ and again at concentrations of 0.27 mg L^−1^ and higher but only in T7 (*p* < 0.05, Figure 2c).

The phase II biotransformation enzyme, GST, and the antioxidant enzyme, SOD, assessed in hepatopancreas were not affected (*p* > 0.05; Figure S1a,b, respectively). Furthermore, no effect was found concerning DNA damage (DNAd) analysed in the hepatopancreas and gills (*p* > 0.05; Figure S1c,d, respectively).

Concerning oxidative stress assessed in muscle, DNAd was significantly reduced in almost all concentrations from both T3 and T7 (*p* < 0.05; Figure 3a), and LPO from muscle had a significant increase in T7 at 0.9 mg L^−1^ (*p* = 0.011, Figure 3b).

The energy metabolism of the green crabs was affected after exposure to sulfoxaflor. The anaerobic and aerobic metabolism was significantly affected in T7 with a LDH decrease at 0.15 mg L^−1^, and an IDH increase at 0.05 mg L^−1^ and a decrease at 0.15 mg L^−1^ (*p* < 0.05, Figure 3c,d, respectively). The LDH/IDH ratio showed a significant reduction at 0.27 mg L^−1^ in T3 and 0.05 mg L^−1^ in T7 (*p* = 0.038; *p* = 0.026, respectively; Figure 3e), as well as a significant difference between exposure time, with a decrease in T7 (*p* = 0.016). Electron transport system (ETS) activity was significantly reduced in all concentrations from both exposure times (*p* < 0.05, Figure 3f), except for the lower concentration in T7.

Sulfoxaflor significantly increased the AChE activity at concentrations 0.08, 0.5 and 0.9 mg L^−1^ in T3 (*p* = 0.048, *p* = 0.006 and *p* = 0.026, respectively). No differences were found in T7 (*p* > 0.05, Figure 3g).

### 3.3. Integrative Analysis of Behavioural and Biochemical Responses

**Figure 4:**
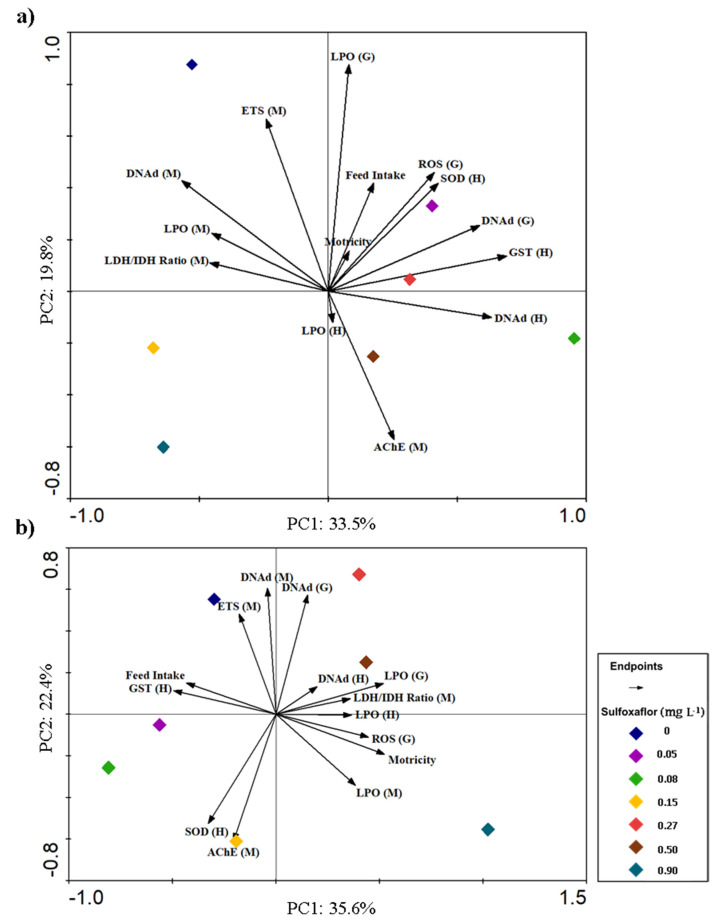
Principal component analysis (PCA) of biochemical/behavioural data average and sulfoxaflor concentrations on different times of exposure: (**a**) exposure time 3 days; (**b**) exposure time 7 days. Diamonds represent different sulfoxaflor concentrations applied in sub-lethal assays, and arrows indicate all types of behavioural responses (feed intake and motricity) and biomarkers assessed. From gills: ROS = reactive oxygen species; DNAd (G) = DNA damage; LPO (G) = lipid peroxidation. From hepatopancreas: GST = glutathione S-transferase; SOD = superoxide dismutase; DNAd (H) = DNA damage; LPO (H) = lipid peroxidation. From muscle: LDH/IDH ratio = lactate dehydrogenase/isocitrate dehydrogenase ratio; ETS = electron transport system; DNAd (M) = DNA damage; LPO (M) = lipid peroxidation; AChE = acetylcholinesterase.

To integrate all the responses, two principal component analyses (PCA) were performed, one for exposure time T3 (Figure 4a) and another for exposure time T7 (Figure 4b). For the T3 PCA, the endpoints that contributed the most to explain concentration variability (diamonds) were LPO (G), GST (H), DNAd (M), ETS (M), and AChE (M). LPO (G) was also significantly positively correlated with ROS (G), as could be verified by Spearman’s correlations (*p* < 0.001, r = 0.517; Table S3), meaning that those variables increase or decrease simultaneously with sulfoxaflor exposure. Behavioural responses had minimum contribution in T3 (Figure 4a), with no correlations between them (Spearman, *p* > 0.05), while their influence increased in T7 (Figure 4b), mainly due to motricity, which at this point is negatively correlated with feed intake (*p* = 0.026, r =−0.318; Table S3). In fact, in the T7 PCA, motricity is associated with several endpoints, being the positive association with LPO (M) significantly correlated (Spearman, *p* = 0.023, r = 0.324, Table S3). As in T3, the positive correlation between ROS (G) and LPO (G) continues to be verified at T7 (*p* < 0.001, r = 0.562). Moreover, in T7 PCA, it is possible to observe a clear separation along PC1 axis between the lowest and the highest concentrations. The three highest concentrations are mainly characterized by lower detoxification capacity (GST), higher LPO levels, and effects on behaviour (higher motricity and lower feed intake).

## 4. Discussion

Neonicotinoids reported mode of action (MoA) occurs by affecting the nervous system through a continuous stimulation, as a result of the interaction of these chemicals with the nicotinic acetylcholine receptors (nAChR) [61]. Studies have demonstrated that neonicotinoids can affect, directly or indirectly, non-target organisms, such as fish, birds, mammals [62], and also aquatic invertebrates [61]. In this context, the bumblebee *Bombus terrestris* exposed to sulfoxaflor revealed sub-lethal adverse effects, by producing less workers and consequently producing fewer reproductive offspring [12] and leading to reduced egg laying [63]. Additionally, according to the technical bulletin of Dow AgroSciences [8] and Niesen et al. [64], sheepshead minnows (*Cyprinodon variegatus*) had reduced growth when exposed to sulfoxaflor. Furthermore, this insecticide is considered highly toxic to mysid shrimp (*Americamysis bahia*) [64], and recent studies have demonstrated sulfoxaflor toxicity to the zebrafish *Danio rerio* [65,66]. Still, few studies have demonstrated sulfoxaflor effects on non-target estuarine/marine organisms.

The current study investigated acute and sub-lethal toxicity on adult male *Carcinus maenas* exposed to sulfoxaflor, demonstrating distinct effects on different levels of biological organisation. A 96 h LC_50_ of 35.13 mg L^−1^ and of 266 mg L^−1^ were found for the fish *Danio rerio* and *Cyprinodon variegatus*, respectively [64,66], while for crustaceans a 96 h LC_50_ of 0.64 mg L^−1^ was found for *Americamysis bahia* [64] and 2.88 mg L^−1^ for *C. maenas* (present study). These latter lower LC_50_ suggest that sulfoxaflor presents higher acute toxicity to marine crustaceans than freshwater/marine fish.

*Carcinus maenas* exposure to sulfoxaflor resulted in a prolonged time to reach the food and a lower feed intake. Food consumption rate has been used to evaluate *C. maenas* behaviour after exposure to contaminants [43,67]. Sulfoxaflor acts in the nervous system of organisms through interaction with nAChR and this MoA may be responsible for the behavioural detrimental responses in *C. maenas*, similar to those observed in the aphid *Myzus persicae* related to excitatory symptoms induced by sulfoxaflor [4,11]. A negative correlation between feed intake and motricity (T6) was obtained in spearman correlation and observed in PCA analysis, meaning that with increasing concentrations of sulfoxaflor, there is a tendency for longer time to reach food and for a lower food intake, showing a pronounced behavioural impact, which can alter their fitness and chances of survival in natural ecosystems [68].

Previous studies have reported that the neurotransmission enzyme acetylcholinesterase (AChE) is the main cholinesterase form available in *C. maenas* muscle, as seen in Rodrigues et al. [69]. A recent publication has demonstrated that sulfoxaflor affected AChE in *Danio rerio* [66], increasing its activity in brain and muscle tissues, despite not presenting a dose-dependent response. In the present study, *C. maenas* exposed to sulfoxaflor also demonstrated a significant increase in AChE after 3 days of exposure (T3) and presented no significant responses after 7 days of exposure (T7). Several studies reported AChE activity increase after exposure to different contaminants, e.g., Reddy and Venugopal [70], Silva et al. [71], and Qi et al. [72]. The increased AChE activity, after exposure to sulfoxaflor, may be related to sulfoxaflor’s binding to the receptor acting as an agonist chemical, causing excitatory responses to the organisms. It is expected that sulfoxaflor competes with acetylcholine for the nAChR receptor [73]. Thus, it is reasonable that in the range of concentrations applied in this study, the organisms might be accumulating AChE in the synaptic cleft due to increased and continuous agonist-based neuromuscular stimuli and to ACh accumulation in this cleft.

Regular cellular functions commonly produce ROS, while xenobiotics can potentiate its production and induce toxicity in aquatic organisms, as reviewed in Livingstone [74]. According to previous studies, neonicotinoids have demonstrated an imbalance in the antioxidant system in aquatic organisms, and induce oxidative damage correlated with a high production of ROS [75,76,77]. A set of defence mechanisms can be activated to remove these oxyradicals, such as antioxidant enzymes and low molecular weight scavengers [22], thus avoiding oxidative damage in the organisms [21]. SOD and GST are some of the enzymes involved in the process of defence against oxyradicals and in detoxification mechanisms [26,27]. Despite no significant responses on these enzymes’ activities were found upon exposure to sulfoxaflor, in the T7 PCA for the higher concentrations, the decrease of GST relates to increasing LPO levels. The GST failure to detoxify, and even the inability of SOD to remove ROS, may lead to an increase in LPO levels at the highest concentration. Benli and Çelik [65] found that sulfoxaflor significantly reduced GST activity in *Danio rerio* gills after 96 h of exposure at concentrations 1.75 and 3.51 mg L^−1^.

Besides these enzymatic observations, a significant reduction of oxidative damage was detected in LPO (G) and DNAd (M), mainly in T3 exposure time. Given this, one cannot exclude that other antioxidant and/or detoxification enzymes not addressed in this study may be activated as a fast response to the initial stage of exposure and induce an overcompensation in the first instance. However, these lower damage effects are most probably derived from lower activity and, consequently, lower respiration rates, thus reducing their metabolism, which also naturally reduces ROS formation. This hypothesis is further supported by the significant reduction in ETS activities at almost all sulfoxaflor concentrations, which may in fact point towards an activity reduction in a very early and low concentration stage, which may influence the myriad of responses addressed in this study, making their interpretation increasingly complex. However, further in time, and due to continuous exposure, there is already evidence of oxidative damage, as reported for LPO in hepatopancreas and muscle. Benli and Çelik [65] also reported increases in TBARS levels after 96h exposure to sulfoxaflor in zebrafish.

The electron transport system (ETS) is a metabolic energy biomarker related to the organism oxygen consumption [32]. In the present study, sulfoxaflor induced a significant decrease in ETS in almost all concentrations from both exposure times, thus revealing a decrease in cellular metabolism. In this context, the reduced ETS activity may be a consequence of organism damaging mechanisms such as oxidative stress, since lipid peroxidation may also exist in the inner mitochondria membrane, damaging it, and consequently impairing metabolism and causing an ETS reduction [78,79]. Therefore, the LPO damage assessed in some tissues of *C. maenas* after exposure to sulfoxaflor may influence mitochondria membrane damage, thus altering ETS functions and reducing its activity (as can be seen in PCA T7).

The organism’s physiological reaction to stressors is due to its association with the changes in biomarkers endpoints as, for instance, a decrease in behavioural activity could imply a reduced respiration and ETS reduction in this study, which may then greatly limit the oxidative damage demonstrated herein. The interaction between sulfoxaflor concentration magnitude and behavioural responses may underlie the greater difficulty in linking data. Thus, for a better understanding, overview, and interconnection among behavioural and biochemical biomarkers, future studies should include physiological processes, for instance, heart-rate, respiration rates, growth, and reproduction [15,80]. Despite further increasing the complexity of endpoints, the present inclusion of several tissues and sampling times contributes to enlighten response interconnection, complexity, and challenge non-monotonicity.

## 5. Conclusions

The present study demonstrated that exposure to sulfoxaflor can affect the non-target coastal invertebrate *Carcinus maenas* at different levels of biological organisation, with its increasing concentrations, which causes a decrease in detoxification capacity and an increase in oxidative damage, such as LPO, while reductions in energy metabolism were also noticed. Additionally, an impact on motricity and feeding was pronounced. These findings highlight sub-individual endpoints to act as early warning signs that could lead to later and higher biological level effects [81], namely upon exposure to xenobiotic. These integrative approaches deepen the understanding of the potential detrimental effects of contaminants in organisms, and on forthcoming impacts at higher levels of biological organisation. Despite the lack of information related to monitoring sulfoxaflor concentrations in the aquatic environment, detrimental effects were observed for *C. maenas* at concentrations lower than those found for other studied organisms. Since there are few in vivo studies on the effects of sulfoximine class on non-target species, this study contributes to increase the knowledge on the insecticide sulfoxaflor mode of toxic action and its impacts on coastal/marine invertebrates. Still, additional studies are needed to better comprehend sulfoxaflor’s mode of action on crabs and other invertebrates, providing further information on the environmental risks caused by this insecticide in coastal regions where it may be easily found.

## Data Availability

On reasonable request, all data can be received from the corresponding authors.

## Supplementary Materials

The following are available online at https://www.mdpi.com/article/10.3390/biology10121234/s1. Figure S1. Biochemical biomarkers responses measured in hepatopancreas and gills of *Carcinus maenas* after 3 and 7 days of exposure to sulfoxaflor (mean ± SD), related to detoxification—(a) glutathione S-transferase (GST) measured in hepatopancreas, oxidative stress (b) superoxide dismutase (SOD) and (c) DNA damage (DNAd) measured in hepatopancreas; and (d) DNAd measured in gills. * Indicates statistically significant differences in relation to control, and # denotes statistically significant differences between exposure time 3 and 7 days (*p* < 0.05, GzLM, followed by pairwise comparison method with adjustment to LSD). Only the biomarkers with no significant differences are shown, compared to control, Table S1. Hydrological parameters (mean ± SD) assessed during acute and sub-lethal tests: temperature; salinity; pH; and DO = dissolved oxygen. Table S2. Estimated lethal concentration (LCx) for *Carcinus maenas* after 96h exposure to sulfoxaflor. Table S3. Spearman’s correlation among all biomarkers and behavioural responses. Positive or negative significant correlation are highlighted in bold and with asterisks (* *p*-value < 0.05, ** *p*-value < 0.01). Time of exposure to sulfoxaflor—T3 = exposure time 3 days; T6 = exposure time 6 days; T7 = exposure time 7 days. Behavioural endpoints assessed—feed intake; motricity. Tissues analysed—(G) = gills; (H) = hepatopancreas; (M) = muscle. Detoxification biomarkers—GST = glutathione S-transferase, oxidative stress—SOD = superoxide dismutase; ROS = reactive oxygen species; DNAd = DNA damage; LPO = lipid peroxidation, energy metabolism—IDH = isocitrate dehydrogenase; LDH = lactate dehydrogenase; ETS = electron transport system, and neuromuscular toxicity- AChE = acetylcholinesterase.
