# Supplementary material for: Exposure to the Insecticide Sulfoxaflor Affects Behaviour and Biomarkers Responses of Carcinus maenas (Crustacea: Decapoda)
Source: Biology (Basel). 2021 Nov 26;10(12):1234. doi: 10.3390/biology10121234 (PMC8698531; doi:10.3390/biology10121234)
Supplement: Supplementary file 1 [file biology-10-01234-s001.zip › biology-1444375-supplementary.pdf]

Supplementary Material

Exposure to the insecticide sulfoxaflor affects behaviour and biomarkers responses of *Carcinus maenas* (Crustacea: Decapoda)

Jadilson M. Damasceno, Lénia D. Rato, Tiago Simões, Inês F. C. Morão, Gabriela Meireles, Sara C. Novais, Marco F.L. Lemos

Hydrological parameters assessed every day throughout the experiment

Table S1. Hydrological parameters (mean ± SD) assessed during acute and chronic tests: temperature; salinity;.pH; and DO = dissolved oxygen.

Water Parameters	Target parameters	Acute assay	Chronic assay
Temperature (°C)	19	19.21 ± 0.07	18.95 ± 0.04
Salinity (ppt)	34	33.89 ± 0.10	34.10 ± 0.01
pH (NBS Scale)	7.9 – 8.2	7.87 ± 0.11	8.07 ± 0.08
DO (mg.L^{-1})	6 - 8	5.67 ± 0.52	6.37 ± 0.11

Figures, tables and statistical analysis results

Table S2. Estimated lethal concentration (LCx) for *Carcinus maenas* after 96h exposure to sulfoxaflor.

		95% Confidence Interval ($mg.L^{-1}$)	
LC level	Concentration ($mg.L^{-1}$)	Lower	Upper
0.20	4.00	3.09	7.52
0.25	3.75	2.91	6.55
0.30	3.53	2.74	5.81
0.35	3.35	2.59	5.22
0.40	3.18	2.44	4.74
0.45	3.02	2.29	4.34
0.50	2.88	2.14	4.00
0.55	2.74	1.99	3.71
0.60	2.61	1.83	3.46
0.65	2.47	1.68	3.23
0.70	2.34	1.52	3.03
0.75	2.21	1.35	2.85
0.80	2.07	1.18	2.67
0.85	1.92	1.01	2.49
0.90	1.74	0.82	2.30

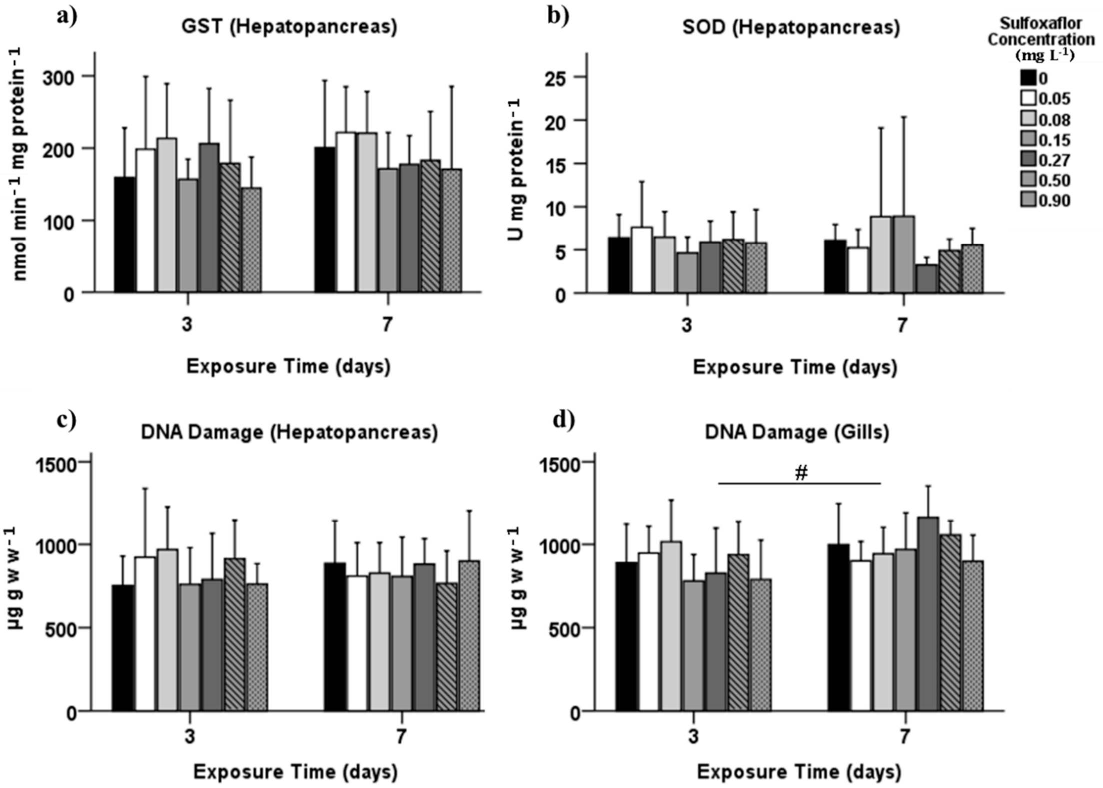

Figure S1. Biochemical biomarkers responses measured in hepatopancreas and gills of *Carcinus maenas* after 3 and 7 days of exposure to sulfoxaflor (mean ± SD), related to detoxification - a) glutathione S-transferase (GST) measured in hepatopancreas, oxidative stress – b) superoxide dismutase (SOD) and c) DNA damage (DNAd) measured in hepatopancreas; and d) DNAd measured in gills. * Indicates statistically significant differences in relation to control, and # denotes statistically significant differences between exposure time 3 and 7 days ($p < 0.05$, GzLM, followed by pairwise comparison method with adjustment to LSD). Only the biomarkers with no significant differences are shown, compared to control.

Table S3. Spearman's correlation among all biomarkers and behavioural responses. Positive or negative significant correlation are highlighted in bold and with asterisks (* p-value < .05, ** p-value < .01). Time of exposure to sulfoxaflor - T3 = exposure time 3 days; T6 = exposure time 6 days; T7 = exposure time 7 days. Behavioural endpoints assessed - feed intake; motricity. Tissues analysed - (G) = gills; (H) = hepatopancreas; (M) = muscle. Detoxification biomarkers - GST = glutathione S-transferase, oxidative stress - SOD = superoxide dismutase; ROS = reactive oxygen species; DNAd = DNA damage; LPO = lipid peroxidation, energy metabolism - IDH = isocitrate dehydrogenase; LDH = lactate dehydrogenase; ETS = electron transport system, and neuromuscular toxicity- AChE = acetylcholinesterase.

	Feed Intake T3	Motricity T3	ROS T3 (G)	DNAd T3 (G)	LPO T3 (G)	GST T3 (H)	SOD T3 (H)	DNAd T3 (H)	LPO T3 (H)	AChE T3 (M)	IDH T3 (M)	LDH T3 (M)	LDH/IDH Ratio T3 (M)	ETS T3 (M)	DNAd T3 (M)	LPO T3 (M)	Feed Intake T6	Motricity T6	ROS T7 (G)	DNAd T7 (G)	LPO T7 (G)	GST T7 (H)	SOD T7 (H)	DNAd T7 (H)	LPO T7 (H)	AChE T7 (M)	IDH T7 (M)	LDH T7 (M)	LDH/IDH Ratio T7 (M)	ETS T7 (M)	DNAd T7 (M)	LPO T7 (M)
**Feed Intake T3**																																
**Motricity T3**	-0,260																															
**ROS T3 (G)**	0,079	0,262																														
**DNAd T3 (G)**	0,109	-0,173	0,133																													
**LPO T3 (G)**	0,242	0,066	**,517****	**,360***																												
**GST T3 (H)**	0,026	-0,037	0,109	0,118	0,138																											
**SOD T3 (H)**	-0,046	-0,013	0,063	-0,096	-0,025	-0,171																										
**DNAd T3 (H)**	-0,057	-0,185	-0,008	**,373****	0,116	-0,108	0,027																									
**LPO T3 (H)**	0,057	0,175	0,137	-0,004	**,334***	**,378****	**-,421****	-0,133																								
**AChE T3 (M)**	-0,203	0,167	-0,055	-0,062	-0,092	-0,137	0,199	0,200	-0,049																							
**IDH T3 (M)**	0,101	0,049	**,329***	0,202	0,204	**,290***	-0,272	-0,025	**,297***	0,084																						
**LDH T3 (M)**	-0,026	0,005	0,061	0,046	0,097	0,164	**-,418****	0,073	**,419****	-0,263	0,188																					
**LDH/IDH Ratio T3 (M)**	-0,081	-0,046	**-,290***	-0,158	-0,107	-0,131	-0,131	0,076	0,098	-0,218	**-,670****	**,549****																				
**ETS T3 (M)**	-0,124	-0,091	-0,099	0,191	-0,011	-0,054	-0,044	0,176	-0,183	0,027	-0,014	0,116	0,110																			
**DNAd T3 (M)**	-0,200	-0,160	-0,102	-0,115	0,023	-0,007	-0,067	0,007	0,034	-0,242	-0,152	**,420****	**,478****	0,139																		
**LPO T3 (M)**	0,261	0,111	0,155	-0,214	0,245	-0,040	0,158	-0,172	0,067	-0,001	-0,097	-0,217	-0,075	-0,180	0,075																	
**Feed Intake T6**	**,353***	-0,125	-0,102	-0,043	-0,067	0,007	-0,077	-0,005	0,060	-0,261	0,273	0,126	-0,177	-0,096	-0,023	-0,106																
**Motricity T6**	-0,092	0,104	0,072	-0,179	-0,122	0,192	0,057	-0,183	0,053	0,064	0,074	-0,129	-0,124	-0,228	-0,051	0,015	**-,318***															
**ROS T7 (G)**	-0,126	-0,067	**-,329***	-0,263	**-,287***	**-,290***	-0,014	0,103	-0,020	0,073	-0,216	0,129	**,327***	**,324***	**,352***	-0,001	0,052	-0,007														
**DNAd T7 (G)**	-0,048	0,131	0,100	0,086	0,088	0,015	0,024	0,235	-0,024	0,060	-0,031	-0,047	0,015	0,211	0,023	-0,014	-0,083	0,107	0,053													
**LPO T7 (G)**	-0,250	-0,159	-0,104	-0,070	0,017	-0,020	-0,024	0,207	0,041	-0,012	0,038	0,254	0,196	0,127	**,359***	-0,064	-0,096	0,151	**,562****	0,165												
**GST T7 (H)**	-0,008	0,242	**,294***	0,079	**,374****	-0,106	0,097	-0,031	0,036	-0,002	0,234	-0,112	**-,313***	-0,048	-0,144	0,048	0,051	-0,141	-0,212	-0,170	**-,286***											
**SOD T7 (H)**	-0,162	-0,122	-0,091	-0,072	-0,152	-0,200	**,294***	-0,184	-0,187	0,120	-0,208	-0,242	-0,043	0,035	0,025	0,084	-0,250	0,014	0,092	-0,263	-0,083	0,109										
**DNAd T7 (H)**	0,209	-0,062	0,063	0,047	0,133	0,256	-0,025	-0,188	0,008	-0,073	-0,060	-0,054	0,038	-0,126	0,046	0,067	-0,094	0,154	**-,320***	0,139	-0,171	**-,332***	-0,173									
**LPO T7 (H)**	0,081	0,153	0,047	-0,002	0,028	0,048	-0,261	0,121	0,184	0,106	0,179	-0,012	-0,133	-0,001	**-,370****	-0,116	0,083	0,171	0,035	0,146	0,019	0,090	**-,434****	-0,007								
**AChE T7 (M)**	0,133	-0,261	-0,062	-0,011	-0,159	-0,032	0,009	0,052	0,046	-0,072	0,124	0,089	-0,087	-0,093	0,037	0,158	0,191	0,153	-0,225	-0,079	-0,210	-0,179	0,201	0,111	-0,216							
**IDH T7 (M)**	0,160	-0,182	0,187	0,124	0,143	0,045	0,221	0,248	-0,229	**-,290***	0,083	-0,041	-0,159	-0,054	0,024	0,088	0,137	0,009	-0,166	0,124	0,079	0,016	-0,097	0,164	0,080	0,175						
**LDH T7 (M)**	0,044	0,171	0,227	0,008	0,208	0,086	0,034	0,074	0,009	-0,017	0,136	-0,047	-0,147	0,123	-0,092	0,067	-0,105	-0,005	-0,173	0,046	-0,244	0,240	-0,225	0,118	0,201	0,028	**,425****					
**LDH/IDH Ratio T7 (M)**	-0,144	**,327***	0,106	-0,111	0,138	-0,024	-0,213	-0,142	0,265	0,167	-0,065	0,123	0,171	0,093	-0,016	0,051	-0,251	0,086	-0,020	-0,141	-0,198	0,167	-0,115	-0,130	-0,038	-0,034	**-,544****	**,345***				
**ETS T7 (M)**	0,148	-0,192	0,134	0,061	0,073	-0,199	0,020	0,259	-0,090	-0,072	0,048	-0,121	-0,145	0,062	0,139	0,236	0,109	-0,081	0,000	0,281	0,100	-0,042	0,005	0,009	-0,037	0,072	**,579****	0,257	**-,352***			
**DNAd T7 (M)**	-0,116	0,167	0,101	0,009	0,176	0,024	0,050	0,091	-0,156	0,025	-0,039	-0,171	-0,123	**,417****	0,034	0,042	-0,095	-0,190	-0,063	0,254	-0,065	0,013	-0,204	**,298***	0,086	-0,200	**,289***	**,580****	0,095	**,368****		
**LPO T7 (M)**	-0,123	0,047	-0,179	**-,292***	**-,406****	0,020	0,001	**-,335***	0,038	-0,163	0,091	0,157	0,050	0,010	0,254	-0,049	0,182	**,324***	0,188	-0,176	0,189	-0,279	0,138	-0,001	-0,008	**,306***	-0,024	**-,307***	-0,217	-0,079	-0,173	
